# Carbohydrate dose influences liver and muscle glycogen oxidation and performance during prolonged exercise

**DOI:** 10.14814/phy2.13555

**Published:** 2018-01-15

**Authors:** Andy J. King, John P. O'Hara, Douglas J. Morrison, Tom Preston, Roderick F. G. J. King

**Affiliations:** ^1^ Research Institute for Sport Physical Activity and Leisure Leeds Beckett University Leeds United Kingdom; ^2^ Scottish Universities Environmental Research Centre East Kilbride United Kingdom

**Keywords:** Carbohydrate ingestion, exercise, metabolism, muscle glycogen, stable isotope

## Abstract

This study investigated the effect of carbohydrate (CHO) dose and composition on fuel selection during exercise, specifically exogenous and endogenous (liver and muscle) CHO oxidation. Ten trained males cycled in a double‐blind randomized order on 5 occasions at 77% $\dot{V}O_{2\max}$ for 2 h, followed by a 30‐min time‐trial (TT) while ingesting either 60 g·h^−1^ (LG) or 75 g·h^−1^ ^13^C‐glucose (HG), 90 g·h^−1^ (LGF) or 112.5 g·h^−1^ ^13^C‐glucose‐^13^C‐fructose ([2:1] HGF) or placebo. CHO doses met or exceed reported intestinal transporter saturation for glucose and fructose. Indirect calorimetry and stable mass isotope [^13^C] tracer techniques were utilized to determine fuel use. TT performance was 93% “likely/probable” to be improved with LGF compared with the other CHO doses. Exogenous CHO oxidation was higher for LGF and HGF compared with LG and HG (ES > 1.34, *P *<* *0.01), with the relative contribution of LGF (24.5 ± 5.3%) *moderately* higher than HGF (20.6 ± 6.2%, ES = 0.68). Increasing CHO dose beyond intestinal saturation increased absolute (29.2 ± 28.6 g·h^−1^, ES = 1.28, *P* = 0.06) and relative muscle glycogen utilization (9.2 ± 6.9%, ES = 1.68, *P *=* *0.014) for glucose‐fructose ingestion. Absolute muscle glycogen oxidation between LG and HG was not significantly different, but was *moderately* higher for HG (ES = 0.60). Liver glycogen oxidation was not significantly different between conditions, but absolute and relative contributions were *moderately* attenuated for LGF (19.3 ± 9.4 g·h^−1^, 6.8 ± 3.1%) compared with HGF (30.5 ± 17.7 g·h^−1^, 10.1 ± 4.0%, ES = 0.79 & 0.98). Total fat oxidation was suppressed in HGF compared with all other CHO conditions (ES > 0.90, *P *=* *0.024–0.17). In conclusion, there was no linear dose response for CHO ingestion, with 90 g·h^−1^ of glucose‐fructose being optimal in terms of TT performance and fuel selection.

## Introduction

During prolonged exercise, endogenous carbohydrate (CHO) and fat act as metabolic substrates to provide energy to the working muscle (Van Loon et al. 2001), with both intra and extra‐myocellular sources essential for strenuous exercise. The protection of finite reserves of liver and muscle glycogen, by provision of exogenous sources of CHO just before and during exercise, may contribute to a prolonged exercise performance, especially where a high‐intensity effort toward the end of exercise is required. However, evidence of “sparing” glycogen with exogenous CHO feeding has received equivocal support (Stellingwerff and Cox 2014), indicating the mechanisms behind the ergogenic benefit of CHO ingestion are likely multi‐factorial, but also sensitive to dose of CHO ingested (Wallis et al. 2007; Smith et al. 2013).

The major rate‐limiting step to exogenous CHO oxidation appears to be the transport of hexoses across the intestinal mucosa. Due to saturation of the glucose specific sodium dependent glucose transporter (SGLT1), glucose has an upper oxidation rate of ~1.0–1.1 g·min^−1^ during exercise (Jentjens et al. 2004b), whereas the addition of fructose can increase this to ~1.6–1.8 g·min^−1^ by exploiting the glucose transporter‐5 (GLUT‐5) intestinal transporter (Adopo et al. 1994; Jentjens et al. 2004a; Jeukendrup et al. 2006). Therefore, to increase exogenous, and attenuate endogenous CHO oxidation, it is recommended that athletes ingest multiple transportable CHO (MTC) in the form of glucose and fructose (GF) in a 2:1 ratio exceeding 1 g·min^−1^. However, it should be noted that relative to no CHO ingestion, a positive performance effect can still be achieved with the ingestion of glucose only (Jeukendrup 2014).

Relative to placebo, time to exhaustion has been shown to improve by as much as 30% (Maughan et al. 1996; McConell et al. 1999) and time trial (TT) performance by 5–12% (Hulston and Jeukendrup 2008; Tripplett et al. 2010; Baur et al. 2014) with CHO ingestion. Smith et al. (2010) also found TT performance improved with CHO dose; the largest improvement (10.7%) found with ingestion of 60 g·h^−1^ glucose versus lower 15 and 30 g·h^−1^ doses. However, this CHO ingestion did not exceed the saturation of SGLT1, which appears to be the major rate limiting step to exogenous CHO oxidation (Hawley et al. 1994; Shi et al. 1995). A performance dose response with MTC has received less attention compared with glucose only. Baur et al. (2014) found no further performance benefit by increasing glucose dose from 61.8 g·h^−1^ to 93 g·h^−1^, but a when an isocaloric dose of GF was ingested, TT time was further improved by 1.3%. In the one multiple dose study to investigate MTC ingestion up to and beyond intestinal saturation threshold, Smith et al. (2013) showed the performance response to CHO ingestion may be curvilinear, mathematically deriving an optimum dose for exercise performance of 68–88 g·h^−1^.

Despite evidence that performance is generally enhanced with exogenous CHO, the mechanism of its effect is less clear (Stellingwerff and Cox 2014). Hulston and Jeukendrup (2008) reported improved TT performance with a concurrent reduction in endogenous glycogen oxidation, but conflicting data also exist to this effect (Rowlands et al. 2008; O'Brien and Rowlands 2011). It appears that glycogen sparing is more likely to occur with MTC ingestion (Jentjens et al. 2004a; Pfeiffer et al. 2011), however, to date it seems that reported “whole body” endogenous glycogen sparing may largely be due to changes in liver glycogen oxidation (Gonzalez et al. 2015). Muscle glycogen has been shown to be spared during prolonged running with ingestion of mixed CHO solutions (Tsintzas et al. 1995, 1996) but evidence to this effect is limited to studies quantifying muscle glycogen oxidation with muscle biopsy analysis (Hargreaves et al. 1984; Yaspelkis et al. 1993; Tsintzas et al. 1995, 1996, 2001; Stellingwerff et al. 2007), where the quantification of glycogen oxidation by this method may have some limitations (Constantin‐Teodosiu et al. 1996; Van Thienen et al. 2014).

In the only dose‐dependent investigation using stable isotope tracers to report liver and muscle glycogen oxidation concurrent with performance data, Smith et al. (2010) found a higher glucose dose attenuated liver glycogen oxidation and increased TT performance. However, muscle glycogen oxidation tended to be slightly higher (ES = 0.23) with the highest, 60 g·h^−1^ dose, a potentially undesirable effect for prolonged exercise. Furthermore, this study only investigated CHO doses below the reported upper absorption rate for glucose ingestion, thus potentially not maximizing intestinal CHO transport.

To date, there has been no investigation of a dose response of MTC ingestion on liver and muscle glycogen oxidation and the precise relationship between ingested dose, glycogen sparing and exercise performance requires further elucidation. Therefore, this study investigated the relationship between the dose (60–112.5 g·h^−1^) of ingested CHO (both glucose alone and coingestion of glucose and fructose) on fuel use during 120 min of moderate intensity cycling and subsequent time trial performance. The use of indirect calorimetry combined with ^13^C tracer techniques enabled the estimation of exogenous and endogenous (liver and muscle) contributions to CHO oxidation. It was hypothesized that glucose‐fructose coingestion would improve time trial performance and attenuate endogenous glycogen oxidation to a greater extent than glucose only ingestion.

## Methodology

### Participants

Ten trained, healthy male cyclists volunteered to participate in this study. Participants were required to have trained for >3 times per week in cycling specific training for at least the last 2 years. Mean age, body mass, maximal oxygen uptake ($\dot{V}O_{2\max}$), and maximal power output (*W*_max_) were 30.7 ± 7.9 year, 76.4 ± 9.6 kg, 61.6 ± 7.6 mL·kg^−1^·min^−1^, and 341.3 ± 54.7 W, respectively. Procedures and potential risks were explained before the study and all participants provided written informed consent. The study received institutional ethics approval.

### Preliminary testing

Preliminary testing consisted of two parts; a maximal incremental cycle test to volitional exhaustion to determine *W*_max_ and $\dot{V}O_{2\max}$, and a familiarization effort for the 30‐min time trial used to quantify exercise performance in the subsequent experimental trials. This visit was conducted 1 week before the first experimental trial on a high‐performance ergometer (SRM, Germany). In line with the protocol of Kuipers et al. (1985), an initial intensity of 100 W was completed for 5 min, after which the workload increased by 50 W every 2.5 min until heart rate reached 160 b·min^−1^, after which it increased by 25 W every 2.5 min to volitional exhaustion. *W*_max_ was calculated from:

(1) $$W_{\max}=P_{\mathrm{last}}+P_{\mathrm{end}}\times \left(t/150\right)$$

Where *P*_last_ is the power output (*W*) of the last fully completed increment, *P*_end_ is the power output at volitional exhaustion, and *t* is the time in seconds completed in the last increment. In order to calculate the equivalent power output for the experimental trials, a regression was applied to the incremental test power outputs and the end stage oxygen uptake ($\dot{V}O_{2}$). The power output at the relevant percentage (77%) of $\dot{V}O_{2\max}$ was calculated from the slope and intercept these data according to:

(2) $$\text{Power output at}\;x\%\;\dot{V}O_{2\max}=mx+c$$

Where *m* is the gradient of the $\dot{V}O_{2}$ and power output relationship, *x* the target % of $\dot{V}O_{2\max}$ and *c* the *y* axis intercept of the *r* $\dot{V}O_{2}$ and power output relationship.

$\dot{V}O_{2}$ and carbon dioxide production ($\dot{V}{\text{CO}}_{2}$) measurements were made using an online gas analysis system (Metalyser 3B, Cortex Biophysik GmbH, Germany). The tripleV volume transducer was calibrated using a 3 L syringe (Hans Rudolph Inc., Shawnee, KS, USA), and the gas analyzers were calibrated using a two‐point reference gas calibration with room air and a gravimetric standard gas mixture (BOC gases, Guildford, UK) of oxygen and carbon dioxide in nitrogen (15% O_2_ and 5% CO_2_). The test–retest reliability for $\dot{V}O_{2}$ and $\dot{V}{\text{CO}}_{2}$ (L·min^−1^) had coefficients of variation in 3.32 and 1.33%, respectively.

Following a 20‐min period of active and passive recovery (10 min of each) after the maximal exercise test, participants undertook a 30‐min self‐paced time trial (TT) for familiarization purposes. The objective of the TT was to complete the maximum amount of work possible within 30 min. Participants were given no verbal encouragement during the test, but were able to see their current power output and time completed/remaining.

### Experimental design

Participants completed five experimental trials (separated by 7 days) consisting of 120 min cycling at 77% $\dot{V}O_{2\max}$, followed by a 30‐min self‐paced TT. During each trial participants ingested 250 mL of one of five drinks solutions every 15 min (starting at minute 15 into the exercise protocol). Four CHO solutions, each enriched with 150 mg per 75 g CHO of a universally labeled (U–^13^C_6_) glucose and/or fructose tracer (Sigma Aldrich, St Louis, MO) providing 60 g (LG) and 75 g (HG) of glucose (D‐glucose; Thornton and Ross Ltd, Huddersfield, UK) only, and 90 g (LGF) and 112.5 g (HGF) of glucose and fructose (Danisco, Kettering, UK) (glucose‐fructose ratio 2:1) were prescribed in a randomized, double‐blind design. Furthermore, a placebo trial (PLA) was also conducted to determine the background appearance of ^13^CO_2_ in expired air and the metabolic response without CHO ingestion. All formulations contained 26 mmol·L^−1^ of NaCl (Saxa, Herts, UK), as well as artificial sweetener (aspartame, Morrisons' plc, Bradford, UK) to blind the participants to each condition. The natural *δ* ^13^C abundance of the stock glucose and fructose was measured by isotope ratio mass spectrometry (IRMS, Isoprime, Cheadle, UK), using L‐fucose as an isotopic internal standard as previously described (Morrison et al. 2011). Glucose and fructose were determined to be −25.56‰ and −12.41‰, respectively. The final enrichment of *δ* ^13^C of the ingested CHO solutions was; LG = +146.83 ± 6.29‰, HG = +146.23 ± 5.99‰, LGF = +148.40 ± 5.83‰ and HGF = +147.20 ± 4.27‰. All *δ* ^13^C measurements are quoted with reference to the internationally accepted standard for carbon isotope measurements, Vienna Pee Dee Belemnite (VPDB).

### Diet and physical activity before testing

Participants recorded their food intake and physical activity during the 48 h before the first experimental trial and were instructed to repeat the same diet and activity pattern in the 48 h before subsequent trials. In the 24 h before each experimental trial, participants were required to not undertake any strenuous physical activity and avoid alcohol and caffeine consumption. Furthermore, participants were also asked to undergo an intense training session 48 h before each visit to deplete background levels of ^13^C in glycogen (Harvey et al. 2007). Throughout the experimental trials, participants were asked to refrain from ingesting carbohydrates derived from plants which utilize the C_4_ photosynthetic cycle, in which there is a higher natural abundance of ^13^C (e.g., maize‐derived sugars). Each participant was provided with a list of foods to avoid (Morrison et al. 2000). This precaution ensures that background ^13^CO_2_ abundance was less likely to be perturbed from oxidation of endogenous and dietary substrate stores from naturally “enriched” C_4_ origin. Before each test, a standardized evening meal was consumed 10–12 h before arrival at the laboratory (total, 1443 kcal; 53% CHO, 17% fat, and 30% protein).

### Experimental trials

After a 10–12 h overnight fast, participants reported to the laboratory on each occasion between 0700 and 0900. Upon arrival at the laboratory, an in dwelling catheter (20 gauge Introcan Safety^®^, B. Braun Medical Ltd, Sheffield, UK) was inserted into an antecubital vein for regular blood sampling. Over the next 10‐min resting $\dot{V}O_{2}$ and $\dot{V}{\text{CO}}_{2}$ measurements were made using an online gas analysis system (Metalyser 3B, Cortex, Germany), which was calibrated following the manufacturer's instructions. For the measurement of ^13^CO_2_:^12^CO_2_ in expired air at rest, 12 mL Exetainers (SerCon Ltd, Crewe, UK) of expired gas were collected in duplicate via a mixing chamber (Jaeger, Germany).

Participants then completed 120 min of cycling at 77% $\dot{V}O_{2\max}$ on a high‐performance ergometer. $\dot{V}O_{2}$_,_ $\dot{V}{\text{CO}}_{2}$ and heart rate (HR) were measured every 15 min until the end of exercise. Samples of expired gas for ^13^CO_2_:^12^CO_2_ analysis were collected during the final 60 s of each 15‐minute period. Samples for the analysis of plasma glucose, plasma lactate, serum insulin, serum‐free fatty acids were drawn every 15 min and for ^13^C plasma glucose enrichment at 60, 90, and 120 min. Following each completed 15‐min period of data collection, one of the 250 mL drink solutions was given to the participants, who were instructed to consume the drink as quickly as comfortably possible.

### Analyses

Aliquots of plasma and serum prepared by centrifugation were analyzed for selected metabolites. Glucose (glucose oxidase kit; Instrumentation Laboratory, Monza, Italy, inter assay CV: 5.4%, Intra assay CV: 2.6%) and lactate (Lactate kit, Randox, County Antrim, UK, Inter CV: 4.7%, Intra CV: 2.9%) were analyzed by spectrophotometry (iLab 300 plus, ILab, UK). Insulin was analyzed using a chemoiluminometric immunoassay (ADIVA Centaur, Bayer diagnostics, Berkshire, UK, Inter CV: 3.2–4.6%, Intra CV: 2.6–5.9%). Non‐esterified free fatty acid content of serum was analyzed by an acyl‐CoA synthetase and oxidase assay (NEFA‐HR2, Wako Chemicals GmbH, Germany, Inter assay CV: 1.5%).

The ^13^CO_2_:^12^CO_2_ in expired air was determined by IRMS. The isotopic ratio (^13^C:^12^C) is derived against laboratory CO_2_ (itself calibrated against VPDB) from the ion beam area ratio measurements with correction of the small contribution of ^12^C^16^O^17^O at *m*/*z* 45, (Craig (1957) correction). The ^13^C:^12^C in plasma glucose was determined using LC‐IRMS as described in detail previously (Morrison et al. 2011). Briefly, plasma samples were prepared by ultrafiltration (30,000 molecular weight cut off tubes, Amicon Ultra 4, Millipore, Watford, UK), with an internal standard added (L‐fucose, C_6_H_12_O_5_, Sigma Aldrich) and separated by liquid chromatography to separate the glucose from other constituents prior to “wet‐oxidation” and IRMS analysis of the resulting CO_2_.

### Calculations

Total CHO and fat oxidation (g·min^−1^) were computed from $\dot{V}O_{2}$ and $\dot{V}{\text{CO}}_{2}$ (L·min^−1^) using the stoichiometric equations of Frayn (1983), with protein oxidation during exercise assumed to be negligible.

(3) $$\text{CHO}=\left(4.55\times \dot{V}{\text{CO}}_{2}\right)-\left(3.21\times \dot{V}O_{2}\right)$$

(4) $$\text{Fat}=\left(1.67\times \dot{V}O_{2}\right)-\left(1.67\times \dot{V}{\text{CO}}_{2}\right)$$

The isotopic enrichment of the ingested glucose and fructose, (*R*_exo_), and expired air (*R*_exp_) was expressed in standard *δ* ^13^C units (‰) relative to VPDB (Craig, 1953). Exogenous glucose oxidation derived from glucose and the combined ingestion of glucose and fructose (CHO_EX)_ was computed using the following equation (Peronnet et al. 1990), with the placebo condition establishing the background ^13^CO_2_:^12^CO_2_ during exercise.

(5) $${\text{CHO}}_{\mathrm{EX}}\left(g\cdot {\text{min}}^{-1}\right)=\dot{V}\;{\text{CO}}_{2}\left[\left(R_{\exp}-R_{\mathrm{ref}}\right)/\left(R_{\mathrm{exo}}-R_{\mathrm{ref}}\right)\right]/k$$

Where $\dot{V}\text{CO}$ is in L·min^−1^, *R*_exp_ is the isotopic composition of expired CO_2_, *R*_ref_ is the isotopic composition of expired CO_2_ at the same time point with ingestion of placebo, *R*_exo_ is the isotopic composition of the ingested solution and *k* (0.747 L·g^−1^) is the volume of CO_2_ provided by the complete oxidation of glucose.

Computations were made on the assumption that, in response to exercise, ^13^C is not irreversibly lost in pools of tricarboxylic acid cycle intermediates and/or bicarbonate, and that ^13^CO_2_ recovery in expired gases was complete or almost complete during exercise (Trimmer et al. 2001). Such computation has been shown to underestimate exogenous oxidation rates at the beginning of exercise because of the delay between ^13^CO_2_ production in tissues and its exhalation (Pallikarakis et al. 1991). Therefore, carbohydrate oxidation data are presented for the second hour of the 2 h protocol to allow for a steady‐state condition of ^13^C in the bicarbonate pool to be reached (Robert et al. 1987).

Based on the ^13^C isotopic composition of plasma glucose (*R*_glu_), the oxidation rate of plasma CHO was calculated (Peronnet et al. 1998):

(6) $$\text{Plasma CHO}\left(g\cdot {\text{min}}^{-1}\right)=\dot{V}\;{\text{CO}}_{2}\left[\left(R_{\exp}-R_{\mathrm{ref}}\right)/\left(R_{\mathrm{glu}}-R_{\mathrm{ref}}\right)\right]/k$$

Endogenous CHO oxidation was calculated as the differences between total CHO oxidation and exogenous CHO oxidation. The oxidation rate of muscle glycogen (g·min^−1^), either directly or through the lactate shuttle (Brooks 1986), was calculated by subtracting plasma glucose oxidation from total carbohydrate oxidation (Equation (7)). Finally, the amount of glucose released from the liver was estimated as the difference between plasma glucose and exogenous carbohydrate oxidation (Equation (8)) (Peronnet et al. 1998):

(7) Muscle oxidation=total CHO oxidation−plasma glucose CHO oxidation

(8) $$\begin{array}{c} \text{Liver oxidation = plasma glucose CHO oxidation}-\text{exogenous oxidation} \end{array}$$

### Statistical analyses

The mean value observed for a given variable is presented with the associated standard deviation (mean ± SD) and where comparisons between conditions made as the mean difference with associated confidence limits at the 95% level with Cohen's d effect size [e.g., mean difference, lower limit to upper limit (ES)] as recommended by Hopkins et al. (2009).

In order to provide meaningful terms to the effectiveness of CHO ingestion on exercise performance, a probabilistic magnitude based inference analysis was conducted to analyze the effect of CHO ingestion on the mean power output during the 30‐min TT. Using the coefficient of variation (2.4%) of laboratory cycling TT performance (Hopkins et al. 1999) and the smallest worthwhile change in athletic performance (0.5 × CV) the smallest meaningful effect in power output between conditions was computed to be 1.2%. The effect of CHO ingestion was expressed as a percentage change relative to placebo ingestion following back transformation of the mean of the natural logarithm of the power outputs. The chance that the true value of the effect was larger than the smallest meaningful effect on the 30‐min TT were computed and qualitative terms assigned (Hopkins et al. 2009): <1%, almost certainly not; <5%, very unlikely; <25%, unlikely or probably not; <50%, possibly not; >50%, possibly; >75%, likely or probable; >95%, very likely; >99% almost certain. For non‐performance variables (heart rate, VO_2_, substrate oxidation, and plasma glucose and lactate, serum‐free fatty acid, and insulin concentrations) where a smallest worthwhile change is difficult to calculate, statistical comparisons were also made using a one‐way (dose) or two‐way (dose × time) repeated measures ANOVA with Bonferroni post hoc adjustment (SPSS 20, IBM, New York, USA) as well as Cohen's d effect sizes (ES). ES threshold values were set as 0.2. 0.6, 1.2, 2.0, and 4.0 for small, moderate, large, very large, and extremely large effects, respectively (Hopkins et al. 2009).

## Results

### $\dot{V}O_{2}$, $\dot{V}{\text{CO}}_{2}$ and heart rate

$\dot{V}O_{2}$ and $\dot{V}{\text{CO}}_{2}$ did not significantly differ over time, that is, between the first and second hour of the 2‐h ride (pooled data; ES = 0.16, *P* = 0.8 and *P* = 0.6, ES = 0.07, respectively) or between conditions (*P* > 0.9, ES < 0.24, Table 1). HR increased with time between the first and second hour of the constant load rides in all conditions (main effect *P* = 0.03, ES = 1.4), Table 1. HR was lower for placebo compared with all CHO conditions for both the first (*moderate* effect, ES = 0.69−1.13), and second hour (*large* to *very large* effect, ES = 0.41–0.74) of cycling. However, HR was only significantly lower for placebo compared with HGF during the second hour of cycling (*P* = 0.09, ES = 0.74).

**Table 1:** Respiratory gas exchange, heart rate, and substrate utilization over the first and second hour of the 2 h of exercise at 77% $\dot{V}O_{2\max}$. Data from the first h is presented in the top line of each variable, and the second hour in the bottom line

	Condition				
	Pla	LG	HG	LGF	HGF
HR (b·min^−1^)	146 ± 7	151 ± 8	151 ± 6	152 ± 7	153 ± 6
	154 ± 9^a^	158 ± 11	160 ± 10	158 ± 10	161 ± 10
$\dot{V}O_{2}$ (L·min^−1^)	3.49 ± 0.45	3.61 ± 0.56	3.52 ± 0.58	3.57 ± 0.69	3.61 ± 0.67
	3.62 ± 0.42	3.63 ± 0.49	3.49 ± 0.42	3.62 ± 0.64	3.71 ± 0.63
$\dot{V}{\text{CO}}_{2}$ (L·min^−1^)	3.18 ± 0.41	3.22 ± 0.47	3.19 ± 0.50	3.15 ± 0.62	3.22 ± 0.56
	3.18 ± 0.37	3.23 ± 0.38	3.16 ± 0.36	3.25 ± 0.60	3.31 ± 0.31
RER	0.91 ± 0.04	0.90 ± 0.08	0.91 ± 0.01	0.90 ± 0.03	0.90 ± 0.04
	0.88 ± 0.03	0.90 ± 0.07	0.91 ± 0.01	0.87 ± 0.05	0.90 ± 0.03
CHO_ox_ (g)	186.1 ± 32.7	177.1 ± 31.3	199.1 ± 38.9	182.5 ± 48.8	217.2 ± 40.4
	167.6 ± 30.1	175.5 ± 24.4	187.4 ± 26.1	187.3 ± 35.5	219.1 ± 41.7
Fat_ox_ (g)	36.2 ± 16.4	38.6 ± 20.4	32.4 ± 7.13	36.9 ± 13.9	25.3 ± 8.2
	46.1 ± 13.8	36.5 ± 16.9	36.1 ± 9.4	38.3 ± 11.4	27.6 ± 10.8
Energy expenditure (kCal)	1099.8 ± 182.3	1088.4 ± 151.7	1126.4 ± 201.7	1106.1 ± 220.1	1050.1 ± 206.6
	1117.2 ± 129.3	1097.0 ± 112.6	1115.2 ± 149.1	1132.6 ± 197.8	1187.1 ± 211.6

Data are heart rate in b·min^−1^, $\dot{V}O_{2}$, $\dot{V}{\text{CO}}_{2}$ in L·min^−1^, respiratory exchange ratio (RER), and CHO and fat oxidation in grams. All values are mean ± SD. *N* = 11.

a Denotes PLA significantly lower than HGF.

### Total carbohydrate and fat oxidation

Total energy expenditure was not significantly different between conditions for the 2 h of continuous cycling (PLA = 2217.0 ± 286.5 kCal, LG = 2185.4 ± 259.4 kCal, HG = 2241.5 ± 344.7 kCal, LGF = 2238.7 ± 414.7 kCal, HGF = 2237.2 ± 375.3 kCal; *P* > 0.95, ES < 0.17). In addition, no effects were seen between the first and second hour of exercise *P* > 0.90, ES < 0.22, see Table 1 for data).

Absolute CHO oxidation was not significantly different between conditions (*P* > 0.058) although total CHO oxidation was highest throughout the 2‐h ride (combined first and second hour data) in HGF, producing *moderate* to *large* effect sizes (ES = 0.78–1.37) compared with the other conditions, which were all similar (ES range = 0.22–0.58, Table 1). Absolute CHO oxidation was also highest in HGF during the second hour of cycling, albeit not significantly different between conditions (*P* > 0.06). Total CHO oxidation was *moderately* higher in HGF than LGF (ES = 0.79) and HG (ES = 0.87) and produced *large* effect sizes compared with LG and PLA (ES = 1.22 & 1.37). In contrast, absolute fat oxidation during the 2‐h ride was lower in HGF compared with the other conditions (combined first and second hour data), and remained lowest during the second hour of cycling, despite there being no significant differences between conditions (*P* > 0.192). *Moderate* to *large* effects sizes were observed during the total 2 h ride (ES = 0.76–1.30) and *small* to *large* effects were seen during the second hour of cycling for absolute fat oxidation in HGF compared with the other conditions (ES = 0.46–1.29).

In addition, the relative contribution of total CHO oxidation to the total energy yield (Fig. 2) during the second hour of exercise was significantly higher in HGF compared with PLA (difference = 16.6, 8.7–24.6%, *P* = 0.024, ES = 1.88). Compared with the other CHO conditions, this effect was *moderate* and significant (LG; 9.4, 4.4–14.3%, *P* = 0.049, ES = 0.90. HG; 8.4, 4.5–12.3%, *P* = 0.023, ES = 1.18) or *large* but nonsignificant (LGF; 9.6, 3.1–16.1%, *P* = 0.17, ES = 1.25). The increased relative CHO oxidation in HGF was associated with a concomitant *moderate* (and significant) or *large* (but nonsignificant) reduction in the relative contribution of fat to the energy yield compared with the other CHO conditions (effect sizes, 95% confidence intervals and *P* values the same as reported for CHO).

### δ ^13^CO_2_ in expired gas and δ ^13^C in plasma glucose

**Figure 1:**
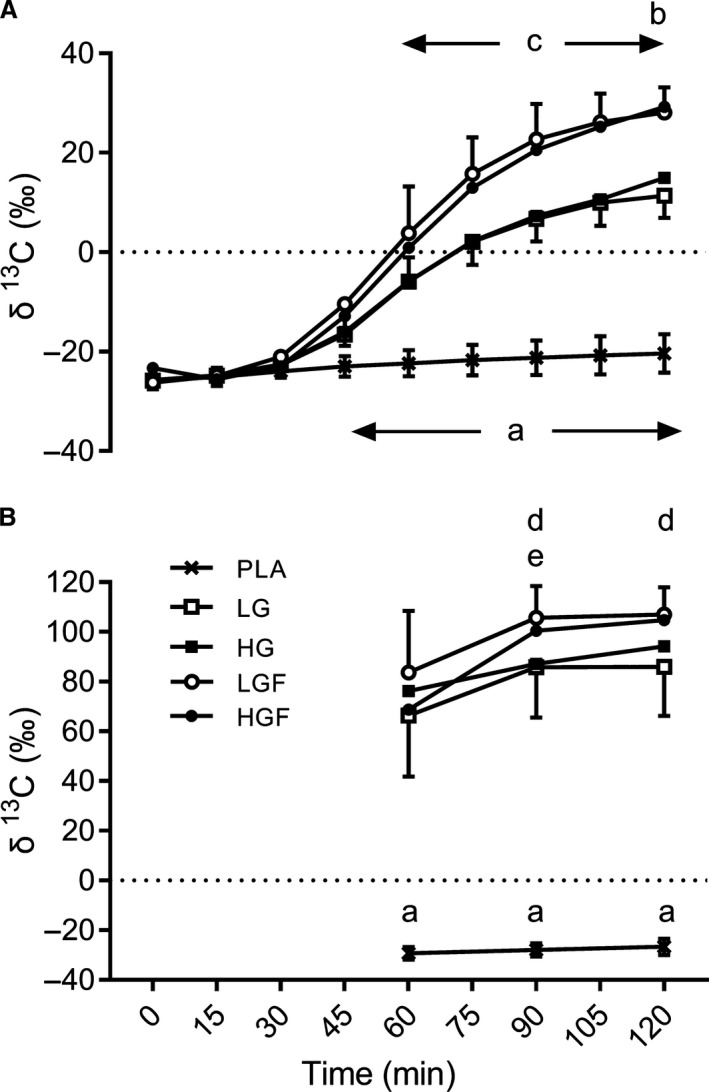
(A) ^13^ CO_2_:^12^ CO_2_ (δ^13^C) in expired air over the 2 h ride and (B) ^13^C:^12^C in plasma glucose during the second hour of the 2 h ride. (a) denotes CHO significantly greater than PLA (*P* = 0.00–0.047), (b) denotes HGF significantly greater than LG & HG (*P* = 0.01 & 0.02), (c) denotes LGF significantly greater than LG & HG (*P* = 0.0–0.024), (d) denotes LGF significantly greater than LG and HG (*P* = 0.026–0.045), (e) denotes 90 min significantly greater than 60 min.

The *δ* ^13^CO_2_ in expired gas was similar between all conditions at rest before exercise and the ingestion of placebo or ^13^C enriched carbohydrate(s) (*P *<* *0.52, ES <  0.50, Fig. 1A). In PLA, the *δ* ^13^CO_2_ in expired gas increased over time by 5.4‰ (ES = 1.95) from the start to the end of exercise. These data were used as the background correction for the calculation of exogenous CHO and plasma glucose oxidation for each CHO condition. The *δ* ^13^CO_2_ in expired gas significantly increased over time from the start of exercise following the ingestion of all four ^13^C enriched CHO conditions (Fig. 1A). Each condition reached maximal values at 120 min, with LGF (28.08 ± 5.12‰) and HGF (29.2 ± 9.5‰) being significantly higher compared with LG (11.35 ± 4.47‰, *P* = 0.001) and HG (14.94 ± 5.97‰; *P* = 0.0005–0.002), with *very large* effects sizes (ES = 1.80–3.48). The *δ* ^13^CO_2_ in expired gas was also significantly higher for LGF and HGF from 60 min onwards compared with LG and HG (*P *<* *0.05, ES = 0.65–3.48).

The isotopic composition of plasma glucose (*δ* ^13^C) increased by 2.6‰ from 60 to 120 min of exercise with ingestion of PLA (*P* = 0.09, ES = 0.9, Fig. 1B). In all CHO conditions, there was a significant rise in plasma *δ* ^13^C glucose between 60 and 90 min (*P* = 0.004–0.012, ES = 1.11–1.40), except in HG where it was *moderate* but nonsignificant (*P* = 0.25, ES = 0.61). Between 90 and 120 min, plasma *δ* ^13^C glucose remained similar for all conditions (*P* > 0.32, ES = 0.11–0.21) except for a *small,* nonsignificant rise in HG (*P* = 1.00, ES = 0.58). The isotopic composition of plasma glucose was highest for LGF during the last hour of exercise, being *moderately* but not significantly higher than LG (66.3 ± 24.4‰, *P* = 0.12, ES = 0.71) and HGF (68.7 ± 26.0‰, *P* = 0.81, ES = 0.60) at 60 min. There were *moderate to large* and significant differences compared with LG at 90 (85.8 ± 20.3‰, *P* = 0.045, ES = 1.17) and 120 min (86.0 ± 19.8, *P* = 0.026, ES = 1.31), as well as HG at 90 (87.2 ± 12.9, *P* = 0.04, ES = 1.44) and 120 min (94.2 ± 11.5, *P* = 0.034, ES = 1.14).

### Sources of oxidized glucose (exogenous and endogenous carbohydrate)

**Figure 2:**
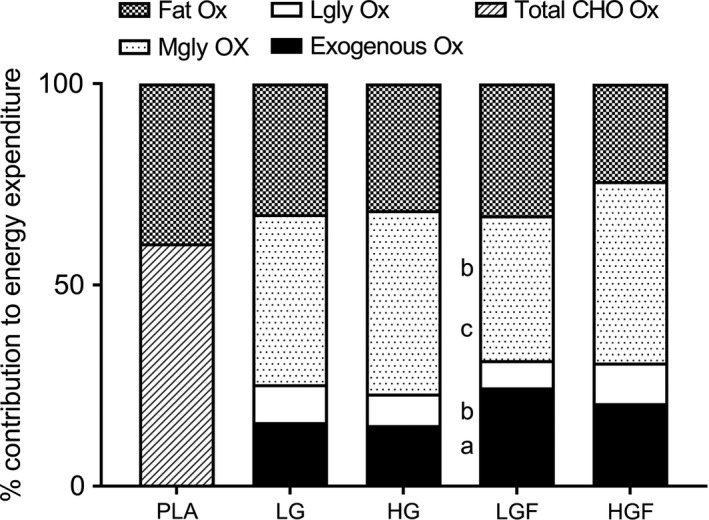
Percentage energy contributions from various substrates during the second hour of the 2 h ride. (a) denotes LGF significantly different to LG (*P* = 0.00), (b) denotes LGF significantly different to HG (*P* = 0.00–0.019), (c) denotes LGF significantly different to HGF (*P* = 0.014)

**Figure 3:**
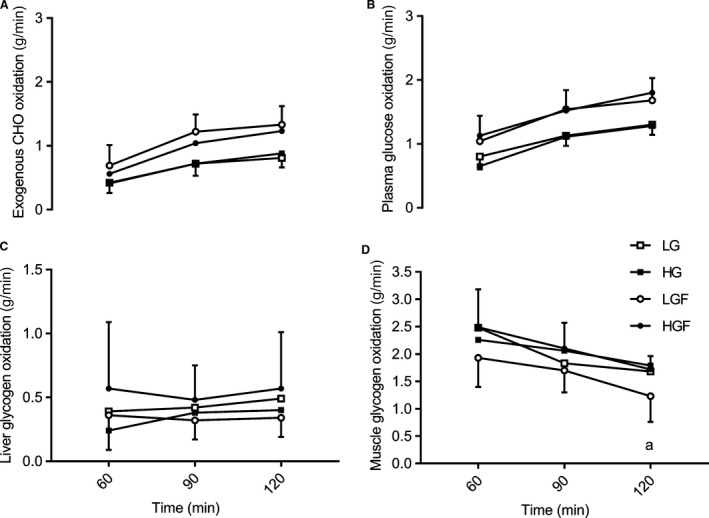
Sources of oxidised glucose and muscle glycogen during the second hour of the ride. A: Carbohydrate from exogenous sources (g.min^−1^) B: Plasma glucose oxidation (g.min^−1^) C & D: Liver and Muscle glycogen oxidation respectively (g.min^−1^) Data are means ± sd. a denotes LGF significantly lower than HG

During the second hour of exercise, the rate of exogenous CHO oxidation was higher with the ingestion of glucose‐fructose than with the ingestion of glucose only (Fig. 3) and was highest with the ingestion of LGF, the highest recorded value occurring after 120 min at 1.33 ± 0.29 g·min^−1^. This was significantly higher, with a *large* effect size compared with both glucose only doses (LG, 0.81 ± 0.15 g·min^−1^; *P* = 0.001, 0.52, 0.37–0.68 g·min^−1^, ES = 2.25, and HG, 0.88 ± 0.23 g·min^−1^; *P* = 0.002, 0.45, 0.30–0.60 g·min^−1^, ES = 1.72). However, when the glucose‐fructose dose was further increased, ingestion of HGF produced a lower *(small* ES*)*, but nonsignificant maximal rate of oxidation (1.23 ± 0.3 g·min^−1^; 0.10, −0.23–0.02 g·min^−1^, *P* = 0.84, ES = 0.36,) compared with LGF. Furthermore, the absolute oxidation of exogenous CHO during the second hour of cycling was significantly higher with glucose‐fructose ingestion compared with glucose only (Table 2; *P* = 0.001–0.037, ES = 1.10–1.87). Total exogenous CHO oxidation in the second hour was also higher with LGF compared with HGF, but this effect was *small* and not significant (see Table 2 for statistical data). Despite there being only a *small* difference between LGF and HGF in absolute terms, the relative contribution of exogenous oxidation to the total energy yield showed a *moderate* and close to significant reduction for HGF compared with LGF during the second hour of exercise (3.7, 1.3 to 6.1%, *P* = 0.06, ES = 0.68). The relative contribution from both glucose‐fructose doses to energy expenditure was significantly greater than with glucose only (8.7, 6.3–11.0% increase between LG & LGF, *P* = 0.001, ES = 1.78; 5.4, 2.5–8.3% increase between HG and HGF, *P* = 0.031, ES = 0.99, Fig.** 2). However, as with the increase in glucose‐fructose dose (LGF to HGF) a linear effect of dose was not observed; increasing dose from LG to HG did not result in an increased energy yield from exogenous CHO (0.7, −1.0–2.4%, *P* = 1.00, ES = 0.16).

**Table 2:** Comparisons of CHO oxidation source during the second hour of exercise at 77% $\dot{V}O_{2\max}$

	CHO_ox_ (g)	Difference in CHO oxidation (g)		
		LG	HG	LGF
Exogenous CHO				
LG	41.2 ± 10.8			
HG	41.4 ± 13.2	0.2, –3.0 to 3.4 ES = 0.02, *P* = 1.00		
LGF	67.7 ± 16.8	26.5, 19.4–33.7 ES = 1.76, *P* = 0.001	26.3, 19.9 to 32.8 ES = 1.87, *P* = 0.001	
HGF	59.2 ± 18.9	18.0, 10.3–25.7 ES = 1.10, *P* = 0.022	17.8, 9.5–26.0 ES = 1.16, *P* = 0.037	−8.5, −14.9 to −2.2 ES = 0.48, *P* = 0.32
Endogenous CHO				
LG	134.3 ± 26.0			
HG	146.0 ± 23.2	11.7, −6.8 to 30.3 ES = 0.48, *P* = 1.00		
LGF	119.6 ± 28.1	−14.7, –35.4 to 6.0 ES = 0.54, *P* = 1.00	−26.4, −39.3 to −13.6 ES = 1.03, *P* = 0.048	
HGF	160.0 ± 33.8	25.7, 8.0–43.3 ES = 0.85, *P* = 0.247	14.0, 4.1 to 23.8 ES = 0.48, *P* = 0.26	40.4, 24.0 to −56.7 ES = 1.30, *P* = 0.017
Plasma glucose				
LG	65.6 ± 9.4			
HG	62.3 ± 14.5	−3.3, –8.8 to 2.2 ES = 0.27, *P* = 1.00		
LGF	87.0 ± 19.1	21.4, 12.2–30.6 ES = 1.43, *P* = 0.024	24.7, 14.1 to 35.4 ES = 1.46, *P* = 0.024	
HGF	89.7 ± 32.1	24.1, 7.2–41.0 ES = 1.02, *P* = 0.26	27.4, 10.4 to 44.3 ES = 1.10, *P* = 0.16	2.7, 9.8–15.1 ES = 0.10, *P* = 1.00
Liver glycogen				
LG	24.4 ± 10.1			
HG	20.9 ± 5.6	−3.5, −8.7 to 1.6 ES = 0.43, *P* = 1.00		
LGF	19.3 ± 9.4	−5.1, −10.6 to 0.3 ES = 0.53, *P* = 0.90	−1.6, −7.9 to 4.7 ES = 0.21, *P* = 1.00	
HGF	30.5 ± 17.7	6.1, −4.6 to 17.0 ES = 0.42, *P* = 1.00	9.6, −1.4 to 20.6 ES = 0.73, *P* = 0.90	11.2, 2.0–20.4 ES = 0.79, *P* = 0.46
Muscle glycogen				
LG	109.9 ± 26.9			
HG	125.1 ± 24.8	15.2, −3.6 to 34.2 ES = 0.60, *P* = 1.00		
LGF	100.3 ± 23.1	−9.6, −27.1 to 8.1 ES = 0.38, *P* = 1.00	−24.8, −37.2 to −12.4 ES = 1.04, *P* = 0.056	
HGF	129.5 ± 22.6	19.6, 6.6 to 32.6 ES = 0.79, *P* = 0.21	4.4, −4.9 to 13.6 ES = 0.18, *P* = 1.00	29.2, 14.3–44.0 ES = 1.28, *P* = 0.062

Values given are comparisons of CHO oxidation from various sources over the second hour of the 2 h ride between LG (60 g·h^−1^), HG (75 g·h^−1^), LGF (90 g·h^−1^) and HGF (112.5 g·h^−1^). [first line: mean ± SD, absolute difference between conditions with associated 95% confidence intervals; second line: Cohen's d effect size and *P* value (ANOVA with Bonferroni post hoc comparison)]. *N* = 11.

Compared with PLA (167.6 ± 30.1 g), CHO ingestion caused a *moderate* reduction in the absolute amount of endogenous CHO oxidation with LG (−33.3, −55.1 to −11.6 g, *P* = 0.32, ES = 1.18) and HG (−21.6, −32.6 to −10.6 g, *P* = 0.10, ES = 0.80), as well as a *large* significant reduction with LGF (−48.0, −67.3 to −28.7, *P* = 0.027, ES = 1.65) during the second hour of cycling. However, the highest dose (HGF) had a similar, and therefore not significantly different, reliance on endogenous CHO oxidation compared with PLA (−7.7, −24.5–9.1 g, *P* = 1.00, ES = 0.24). A linear dose effect was therefore not observed for the absolute amount of endogenous CHO oxidation during the second hour. However, in LGF, endogenous CHO oxidation was significantly lower than HG and HGF and despite not being significantly lower than LG, produced an effect size of 0.54 (10.9% mean reduction) (see Table 2 for comparisons).

The rate of plasma glucose oxidation (Fig. 3B) was not significantly different between conditions during the second hour of exercise. However, despite not being significantly different, the higher rates of plasma glucose oxidation for HGF and LGF compared with HG and LG produced *moderate* differences at 60 min (*P* = 0.076–1.00, ES = 0.59–1.06) and *moderate* or *large* effects at 90 and 120 min (*P* = 0.02–0.25, ES = 1.07–1.68). Differences between LG and HG or LGF and HGF were all *small* and not significant (*P* > 0.65, ES < 0.55). In addition, the absolute contribution of plasma glucose to the total energy yield was not significantly different between LG and HG, with a *small* effect size (Table 2). There were also no significant differences between LGF and HGF. However, the absolute difference in the amount of plasma glucose oxidized was *large* and significantly higher for LGF compared with LG, HG during the second hour of exercise (Table 2).

There were no significant differences in the rate of liver derived glucose between conditions (Fig.** 3C) at any time points. However, at 60 min, HG was *moderately* reduced compared with LG (0.15, −0.28–0.02, *P* = 0.29, ES = 0.92) and HGF (0.33, 0.02–0.64, *P* = 0.41, ES = 0.88). At 90 min LGF was *moderately* reduced compared with HGF (0.16, 0.02–0.30, *P* = 0.32, ES = 0.71,). At the end of the 2‐h ride (120 min), the rate of oxidation of liver derived glucose was lowest with ingestion of LGF (0.34 ± 0.20 g·min^−1^), a *small* but nonsignificant reduction compared to HG (−0.06, −0.15–0.04, *P* = 1.00, ES = 0.39) and a *moderate* nonsignificant reduction compared to LG (−0.15, −0.27 to −0.02, *P* = 0.26, ES = 0.58) and HGF (−0.22, −0.46 to −0.01; *P* = 0.53, ES = 0.68). There were also no significant differences in the absolute oxidation (Table 2), or relative contributions to the total energy yield of liver derived glucose (Fig.** 2) during the second hour of exercise (ES all 0.35–0.93). However, despite not reaching significance, liver glucose oxidation was *moderately* lower in LGF compared with HGF in both the absolute oxidation of liver derived glucose (11.2 ± 17.7, 0.3–22.2 g, *P* = 0.46, ES = 0.79,) and relative contribution to energy yield (3.3 ± 5.0, 0.2–6.4%, *P* = 0.41, ES = 0.93).

Muscle glycogen oxidation reduced throughout the second hour of the 2‐h ride, and was lowest with the ingestion of LGF (Fig.** 3D). In this condition, muscle glycogen oxidation reduced to 1.23 ± 0.5 g·min^−1^ at 120 min, a *moderate*, but nonsignificant effect compared to LG (−0.45, −0.88 to −0.02 g·min^−1^, *P* = 0.43, ES = 0.94), a *large* significant effect to HG (−0.56, −0.86 to −0.26 g·min^−1^, *P* = 0.031, ES = 1.42) and a *large* and nonsignificant effect to HGF (−0.53, −0.87 to −0.19 g·min^−1^, *P* = 0.08, ES = 1.42). Muscle glycogen oxidation rate was also lowest in LGF at 60 (*P* = 0.072–0.97, ES = 0.62–1.02) and 90 min (*P* = 0.76–1.00, ES = 0.32–0.91), with *moderate,* but nonsignificant effects to all CHO doses.

Furthermore, when considered over the whole second hour, absolute muscle glycogen oxidation was lowest in LGF compared with the other CHO conditions, with effects ranging from *small* (8.7% reduction to LG) to *moderate* (19.8% reduction to HG) and *large* (22.5% reduction to HGF), but did not reach significant differences (See Table 2 for confidence intervals and ES). The increased ingestion of both glucose‐fructose and glucose only resulted in higher absolute muscle glycogen oxidation, that is, with glucose‐fructose ingestion above reported intestinal transport saturation, muscle glycogen oxidation was increased and despite not reaching significance, this effect was *large*. Similarly, when the dose of glucose only was increased, absolute muscle glycogen oxidation over the second hour increased. However, this effect was not significant despite producing a *moderate* effect (Table 2).

In terms of the relative contribution to energy yield from muscle glycogen, these effects were magnified. Muscle glycogen oxidation during the second hour of exercise was significantly lower (with *large* effect sizes and significant differences) for LGF compared with HGF (−9.1, −4.9 to −13.4%, *P* = 0.01, ES = 1.68) and HG (−9.6, −13.6 to −5.6, *P* = 0.01, ES = 1.76). Compared with LG, the relative contribution of muscle glycogen oxidation to the energy yield in LGF was also slightly lower, producing a *moderate*, but nonsignificant difference (−6.4, −13.3 to −0.5, *P* = 0.65, ES = 0.73,)

### Circulatory metabolites and insulin

**Figure 4:**
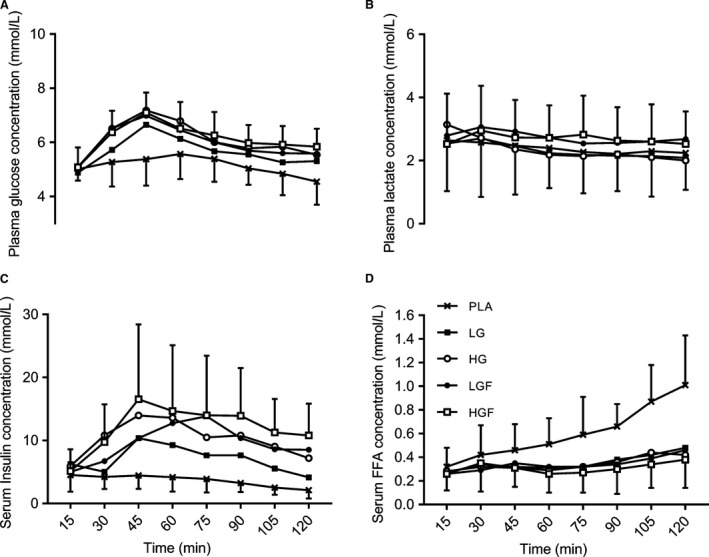
Circulatory metabolites, plasma glucose and lactate, serum‐free fatty acids and insulin concentrations during the 2 h ride. Data are means ± SD. See text for statistical and ES comparisons.

The response of plasma glucose, lactate and serum insulin and FFA throughout the 2‐h ride did not differ significantly between CHO conditions (Fig.** 4). Plasma lactate concentrations after 15 min were similar, that is, not significantly different, across all conditions (2.5 ± 1.6 mmol·L^−1^ to 3.1 ± 2.2 mmol·L^−1^, *P* = 1.00, ES < 0.30) and generally declined slightly, but not significantly, over the 2‐h ride (pooled average change, −0.4, −1.0 to 0.2, *P* = 1.00, ES = 0.29). Compared with the ingestion of glucose only (LG and HG) the ingestion of both glucose‐fructose doses resulted in a *small,* but nonsignificant*,* increase in plasma lactate concentration throughout the 2‐h ride (*P* = 1.00, ES = 0.26 & 0.28).

Plasma glucose concentrations increased in all conditions from the start of exercise, peaking at 45 min in all CHO conditions between 6.6 ± 1.1 and 7.2 ± 1.5 mmol·L^−1^. However, differences were *small* and nonsignificant (*P* = 1.00, ES < 0.46), except in relation to PLA, which was significantly lower (5.4 ± 1.0 mmol·L^−1^; *P* = 0.001–0.05, ES = 1.22–1.99,). Plasma glucose concentration also peaked later in PLA, at 60 min, a *moderate* effect to CHO ingestion, where concentrations were lower than peak values (*P* = 0.02–1.00, ES = 0.49–1.05). From 75 min plasma glucose stabilized, producing *small* effects of time in CHO conditions (*P* = 1.00, ES = 0.52–0.57) and *moderate* nonsignificant effects between the highest and lowest concentrations (HGF vs. LG) at 75 and 120 min (*P* = 0.39 & 0.06, ES = 0.79 & 0.87, respectively). All other comparisons produced *small* nonsignificant effects (*P* = 1.00, ES < 0.54).

Free fatty acid concentrations rose significantly throughout the 2 h ride in PLA (*P* = 0.001, ES = 2.29), reaching a maximum value at the end of exercise (1.01 ± 0.41 mmol·L^−1^). CHO ingestion resulted in significantly lower FFA concentrations compared with PLA, with *large* effects of mean concentration (LG = 0.35 ± 0.17, HG = 0.34 ± 0.12, LGF = 0.36 ± 0.16, HGF = 0.31 ± 0.16) compared with PLA during the 2‐h ride (*P* = 0.002–0.10, ES = 1.21–1.45) and *large* and significant effects at 120 min (*P* = 0.004–0.05, ES = 1.72–1.83). No CHO dose effects were apparent (*P* = 1.00, ES < 0.38).

In PLA, serum insulin steadily declined over the 2‐h ride, but this was not significant (2.41 mU·L^−1^ decrease, *P* = 0.84, ES = 1.09) and was elevated by CHO ingestion (*P* < 0.05, ES > 1.00). After 15 min, serum insulin was similar, that is, not significantly different, between conditions (*P* > 0.18, ES < 0.45) and peaked at 45 min in all CHO conditions, except in LGF (see Fig.** 4). Increasing CHO dose did not alter serum insulin beyond a *moderate,* nonsignificant effect at any time point (ES < 1.01) until the end of exercise where a significant *large* effect was seen between LG and HGF (*P* = 0.01, ES = 1.61,) and *moderate* (nonsignificant) effects between LG and HG & LGF (*P* = 0.50 and 0.53, ES = 0.94 and 0.95).

### Time trial performance

The effect of CHO ingestion was to increase mean power output during the 30‐min time trial compared with placebo (Table 3). With a change in performance of 1.2% used as the smallest meaningful improvement, the ingestion of CHO was at least 81% “likely” to 99% “almost certain” to improve the chance of increasing mean power output. The chances of CHO ingestion causing a detrimental effect were less than 8% “unlikely/probably not” to happen in HG, and <2% “very unlikely” in all other CHO conditions. Significantly, the ingestion of 90 g·h^−1^ glucose‐fructose (LGF) resulted in the highest mean power output, producing a greater than 93% “likely/probable” chance of improved time trial performance compared to the other CHO doses. There was no linear dose relationship between CHO dose and time trial performance, however, and the chances of improving performance by increasing both single and multiple CHO doses were “unlikely” or “very unlikely” beneficial (74% “possible” detrimental effect and a 91% “likely/probably” detrimental effect, respectively for LG vs. HG and LGF vs. HGF).

**Table 3:** Comparison of performance and changes in performance in the 30 minute time trial following 2 h of exercise at 77% $\dot{V}O_{2\max}$

Performance (W)		% Improvement in average power			
		LG	HG	LGF	HGF
Placebo	187 ± 43	11.2, 1.8–21.4 (0.45) 96%, very likely *P* = 0.046	5.1, −2.4–13.1 (0.20) 81%, likely *P* = 0.20	21.1, 9.0–34.5 (0.86) 99%, almost certainly *P* = 0.012	14.9, 5.8–24.7 (0.60) 99%, very likely *P* = 0.015
LG	206 ± 41		−5.5, −16.4–6.8 (0.23) 17%, unlikely *P* = 0.41	8.9, 0.4–18.2 (0.41) 93%, likely, probable *P* = 0.087	3.3, −1.8–8.6 (0.16) 76%, likely *P* = 0.26
HG	196 ± 46			15.2, 2.6–29.4 (0.63) 96%, very likely *P* = 0.064	9.3, −0.9–20.5 (0.38) 91% likely, probable *P* = 0.12
LGF	225 ± 45				−5.1, −0.2–9.8 (0.27) 2%, probably not *P* = 0.11
HGF	213 ± 43				

Data are: mean power outputs during the 30‐minute time trial with ingestion of LG (60 g·h^−1^), HG (75 g·h^−1^), LGF (90 g·h^−1^) and HGF (112.5 g·h^−1^) (mean ± SD). [first line: % difference between conditions following log transformation of data with associated 90% confidence intervals; second line: Cohen's d effect size (in parentheses); third line: chances of (% and qualitative) of meaningful improvement; fourth line: *P* value from ANOVA with LSD post hoc comparison]. *N* = 11.

## Discussion

This study has demonstrated that the ergogenic effect of CHO ingestion during prolonged exercise is sensitive to dose and that the ingestion of 90 g·h^−1^ glucose‐fructose was optimal when compared with lower doses of glucose (60 and 75 g·h^−1^) and a higher dose of glucose‐fructose (112.5 g·h^−1^). Ingestion of 90 g·h^−1^ glucose‐fructose produced the largest oxidation of exogenous CHO which led to an attenuation of pre‐existing muscle glycogen use, with smaller alterations in the oxidation of glucose released from the liver. This has the potential to produce improvements in a subsequent time trial performance following a 2 h fixed load ride compared to the other CHO doses and the placebo. This study also demonstrates an “over‐dose” effect of intestinal CHO transporters for the high dose glucose‐fructose condition compared with one at saturation limits for SGLT1 and GLUT5., There was an increased reliance on liver and muscle glycogen with 112.5 g·h^−1^ of glucose‐fructose, with no further oxidation of exogenous CHO. This was seen with a concurrent reduction in fat oxidation. Therefore, a novel aspect of this study is that the manipulation of CHO dose at and above intestinal CHO transport limits can have a potentially meaningful impact on endogenous fuel use and subsequent time trial performance.

The ingestion of glucose‐fructose resulted in a higher peak rate of exogenous CHO oxidation (1.33 g·min^−1^) than with glucose alone (0.88 g·min^−1^), which is consistent with the existing literature (Jentjens et al. 2005; Smith et al. 2010). This study adds further support for the efficacy of using MTC to maximize exogenous CHO oxidation, (Jentjens et al. 2004a, b; Jeukendrup et al. 2006; Hulston and Jeukendrup 2008; Rowlands and Clarke 2011). As the rate of cellular glucose uptake is not rate limiting in the normal physiological range of plasma glucose concentration with CHO ingestion (Hawley et al. 1994), using MTC maximizes intestinal CHO transport by concurrently using both SGLT1 and GLUT‐5 transport proteins, increasing the availability of CHO for oxidation, which explains the difference in the rates of exogenous CHO oxidation. This study further supports the evidence (Jentjens et al. 2004b, 2006; Jentjens and Jeukendrup 2005; Wallis et al. 2007) that when ingesting glucose or glucose‐fructose, that increasing the dose beyond intestinal saturation of SGLT1 and GLUT‐5 transport proteins provides no further increases in exogenous CHO oxidation. This is likely due to an accumulation of the CHO in the gut (Jeukendrup and Moseley 2010). This delay in CHO absorption across the intestinal lumen may explain the *moderately* reduced relative contribution of exogenous CHO to the total energy yield in HGF compared with LGF when these data are considered for the whole of the second hour of exercise, rather than just the peak oxidation rates. It should be noted that a recent review highlights potential individual variability in exogenous CHO oxidation rates with fructose (co)ingestion, and at ingestion rates approximate or lower than 0.5–0.6 g·min^−1^ for fructose, the ratio of fructose to glucose ingestion may exceed that reported in this study (Rowlands et al. 2015).

The current study also partially supports previous evidence that maintaining high rates of exogenous glucose oxidation, seen with elevated plasma glucose concentrations, may explain the ergogenic benefit of CHO ingestion during prolonged exercise (Coyle et al. 1986; Jeukendrup et al. 2006). In the present study glucose‐fructose ingestion only marginally resulted in higher plasma glucose concentrations than glucose ingestion, but both ingestion of glucose and glucose‐fructose significantly increased plasma glucose concentrations compared with placebo. While plasma glucose concentrations do not reflect rates of glucose flux (i.e., rates of appearance and disappearance of circulatory glucose), the current exogenous CHO glucose oxidation data provide evidence for a greater disposal of glucose into the working muscle in the LGF condition. Therefore, the proposed mechanism of increasing CHO as a substrate to the working muscle may have an upper limit in its effect, as demonstrated by the differing time trial performances with low and high glucose‐fructose conditions.

Small alterations in exogenous CHO oxidation will alter endogenous substrate oxidation where the energy cost of exercise is unchanged. Due to the finite energy resource that glycogen stores provide, it stands that attenuating muscle glycogen use should produce a positive performance benefit, particularly in cycling where power generation is more localized and effective muscle glycogen availability reduced (Arkinstall et al. 2001). This is the first study to show that an increase in glucose‐fructose dose from 90 to 112.5 g·h^−1^ caused a greater reliance (*large* effect) on pre‐existing muscle glycogen. This was associated with a concomitant reduction in oxidation of fat, even though there was no significant difference in absolute exogenous CHO oxidation. However, a *small* difference in the absolute amount of exogenous CHO oxidation (8.5 g) during the last hour of exercise for HGF compared with LGF, could be considered meaningful based on the confidence intervals (Table 2). Therefore, it is interesting that this lower absolute amount of exogenous CHO oxidation was not equivalent to the absolute increase in endogenous glycogen observed between 90 and 112.5 g·h^−1^ (40.4 g, *large* effect); this was predominately from pre‐existing muscle glycogen (*large effect)*, rather than glucose released from the liver (*moderate effect*). This provides evidence from a fuel use perspective, on why individuals should not consume excessively high doses of CHO during endurance exercise, where the availability of CHO may become limited. In addition, when glucose was ingested alone, there was a *moderate* increase in the use of pre‐existing muscle glycogen when the dose was increased above the reported limit for SGLT1, albeit smaller compared with glucose‐fructose. Even though this did not affect fat oxidation, an increase in the use of pre‐existing muscle glycogen for the high dose of glucose is “probably” likely to negatively affect subsequent time trial performance, compared with a dose of glucose at intestinal absorption rates. In addition, glucose‐fructose in comparison with glucose ingestion at the previously reported saturation limits (60 g·h^−1^), was more effective at attenuating muscle glycogen utilization. Even though this effect was *small*, it may be part of the explanation for better performance on LGF compared with LG. Taken together, this leads to the conclusion that in terms of muscle glycogen oxidation, the rate of ingestion should reach, but not exceed, or “over‐dose” intestinal saturation transporters for either glucose (Wallis et al. 2007) or glucose‐fructose use.

These data from the current study shows that muscle glycogen oxidation is increased when CHO ingestion rates exceed intestinal transport rates, which in the case of HGF was associated with a decrease in fat oxidation. This decrease in fat oxidation is not, however, supported by the FFA concentration data, which were similar between conditions during exercise. However, adipose tissue FFA release will likely have been suppressed by the *moderately* elevated insulin concentrations relative to the other conditions (Coyle et al. 1997). The higher circulatory insulin was likely to have inhibited hormone sensitive lipase, suppressing FFA release which is not accounted for in FFA concentrations due to the flux between the circulating substrate and cellular (muscle) uptake. Nevertheless, it is possible that the lack of differences in the FFA concentrations and the *moderately* increased insulin concentrations with 112.5 g·h^−1^ of glucose‐fructose reflect a reduction in IMTG oxidation where carnitine palmitoyl transferase 1 (CPT‐1) and subsequent long chain FA are inhibited (Jeppesen and Kiens 2012). This may in part explain the increased reliance on muscle glycogen as a muscle energy substrate.

The release of glucose from the liver during the 2‐h cycle made a relatively small contribution to the total energy yield compared to that from muscle glycogen. However, the ingestion of 90 g·h^−1^ glucose‐fructose has shown a *moderate* (but nonsignificant) attenuation of liver glycogen oxidation in comparison with 112.5 g·h^−1^, but *small* in comparison with the two glucose doses, which may be important in terms of exercise performance. However, caution is required, as the large individual variation in this study make these data difficult to interpret. It is possible that previous evidence of sparing whole body endogenous glycogen (calculated as the difference between total CHO oxidation measured by indirect calorimetry and exogenous glucose oxidation), (Jentjens et al. 2004a; Jentjens and Jeukendrup 2005; Wallis et al. 2006; Pfeiffer et al. 2011) may be mediated by attenuations in hepatic glucose production, that is, a sparing of liver glycogen, as high CHO doses can dramatically reduce hepatic glucose output (Jeukendrup et al. 1999; Wallis et al. 2006). However, it is noteworthy in this study, that the release of glucose from the liver was highest for 112.5 g·h^−1^ of glucose‐fructose compared with lower doses of glucose and glucose‐fructose. Therefore, further dose response studies are required to establish the optimal effect on liver glycogen oxidation, which has been deemed important for sustaining endurance performance. However, it is worth noting that the dose (~180 g·h^−1^) used in the former of these studies may have excessively over saturated or “over‐dosed” the intestinal SGLT1 transport protein, and are impractical as “real‐world” ingestion rates (Jeukendrup et al. 1999). Liver glycogen sparing has also been seen when just above saturation (102 g·h^−1^;(Gonzalez et al. 2015)) following the ingestion of sucrose. In both studies, it would have been interesting to see whether a lower dose of carbohydrate would have produced better attenuation of liver glycogen oxidation, as observed in this study.

Liver glycogen reserves also form a small contribution to overall glycogen capacity and following an overnight fast, are likely to be in the region of 60 g (Magnusson et al. 1992). Therefore, under the assumption (as the ^13^C isotope tracer methodology cannot reliably determine oxidation rates in the first hour of exercise) that liver glycogen oxidation rates are similar to the second hour, liver glycogen may have been near depletion at the end of exercise in the HGF condition. Whether high rates of liver glycogen oxidation or close to depleted reserves are likely to directly influence exercise capacity in terms of fatigue requires further mechanistic elucidation. However, the association between hypoglycemia and exercise capacity is well established (Coyle and Coggan 1984) with liver glycogen playing an important role in sustaining endurance performance (Casey et al. 2000). It should be noted that the current methodology cannot differentiate between glucose derived from liver glycogen, or glucose derived from gluconeogenic precursors, which may contribute 0.11 ± 0.05 g·min^−1^ of glucose production during prolonged exercise at the lactate threshold in well trained cyclists (Emhoff et al. 2013). It should also be noted that the plasma ^13^C:^12^C with fructose ingestion (i.e., with ^13^C labeled fructose) may be slightly diminished by the “loss” of ^13^C signal due to hepatic conversion of fructose to lactate. Under these possible conditions a number of ^13^C atoms will be attached to newly formed lactate and as such not measured by IRMS. Therefore, it is possible that the subsequently derived muscle and liver glycogen oxidation values will be over and under estimated, respectively. However, this is an area that warrants future research, as recent data suggest that the appearance of circulating lactate is not significantly different between glucose‐fructose and water ingestion. Furthermore, and despite similar plasma lactate concentrations in this study, fructose ingestion can cause an increase in lactate concentrations (LeCoultre et al. 2010) and the rate of appearance of lactate with fructose ingestion during exercise is elevated. However, the methodology used by LeCoultre et al. (2010) cannot apportion the source of lactate production between the possible liver conversion or the conversion of fructose derived glucose which has subsequently undergone oxidation in the contracting muscle.

In this study, improvements in TT performance were not linear with increasing CHO dose. In fact, for both glucose only and glucose‐fructose ingestion, there was an “over‐dose” effect, whereby performance was reduced when CHO doses were ingested in excess of the reported intestinal saturation rates. In this regard, and in agreement with the literature, glucose‐fructose mixtures are more beneficial to prolonged exercise performance (Currell and Jeukendrup 2008; Baur et al. 2014). Whilst dose response literature to this effect is scarce, Smith et al. (2013) showed endurance performance follows a curvilinear response to increasing CHO dose, modeling optimal performance in a 20 km time trial following 2 h of constant load work with 68–88 g·h^−1^ of glucose‐fructose ingestion. However, the study did not report fuel use data and therefore a unique aspect of this study is the finding that the use of pre‐existing muscle glycogen was also attenuated during the 2 h ride with the ingestion of a 90 g·h^−1^ glucose‐fructose solution, which may in part explain the greater mean power output during the subsequent TT in this condition. Therefore, our data also provide evidence to suggest the total rate of exogenous CHO oxidation may not provide a single ergogenic explanation as an additional benefit of “sparing” muscle glycogen with the ingestion of 90 g·h^−1^ glucose‐fructose was also seen.

In conclusion, ingestion of 90 g·h^−1^ glucose and fructose is recommended for prolonged (>2 h) exercise performance due to the potential to spare muscle glycogen oxidation relative to lower doses of single CHO ingestion, and higher MTC ingestion. This falls in line with previous evidence in regards to performance, but provides new insight into the liver and muscle glycogen response to exercise of this nature. Ingesting high amounts of either single or multiple source CHO also creates an “over‐dose” effect, whereby effective and beneficial muscle glycogen oxidation and exercise performance are diminished. Therefore, it should be recommended that the rate of ingestion should reach, but not exceed intestinal saturation for either glucose or glucose‐fructose ingestion. The mechanism(s) behind the ergogenic effect of CHO in endurance exercise are likely more complex than being related to a single factor and data from this study suggest that the effect of CHO ingestion may be both dose dependent and integral to protecting finite glycogen stores which may play a key role in regulating fatigue and prolonged exercise. Further research is warranted to confirm these effects in more prolonged exercise, and to investigate the cellular signaling pathways that to which this effect may be attributable.

## Conflicts of Interest

The authors declare no conflicts of interest. In submission of this paper, the authors declare that the results of the study are presented clearly, honestly, and without fabrication, falsification, or inappropriate manipulation of data.
